# Microbial extraction of rare earth metals: An annotated selection of World Wide Web sites relevant to the topics in microbial biotechnology

**DOI:** 10.1111/1751-7915.14055

**Published:** 2022-03-30

**Authors:** Lawrence P. Wackett

**Affiliations:** ^1^ Department of Biochemistry, Molecular Biology & Biophysics BioTechnology Institute University of Minnesota St. Paul MN 55108 USA

## Rare earth biomining

### https://www.nature.com/articles/s41467‐020‐19276‐w

This paper describes experiments carried out on the International Space Station on leaching of rare earth elements from rocks.

## Microbial extraction of rare earth metals

### https://cen.acs.org/analytical‐chemistry/separations/Microbial‐column‐captures‐rare‐earths/97/web/2019/12

This commentary describes the use of microbes in a hydrogel packed into a column to capture rare earth elements from electronics waste.

## Lanthanide‐dependent methylotrophs

### https://www.ncbi.nlm.nih.gov/pmc/articles/PMC6912076/

This article deals with a specific group of lanthanide‐dependent methylotrophs from the family *Beijerinckiaceae*.

## Methylotrophs and gandolinium

### https://www.biorxiv.org/content/10.1101/2021.06.12.448192v1

The rare earth element gandolinium is used as a magnetic resonance imaging contrast agent and it is increasing in some surface waters. A gandolinium‐dependent methylotroph was examined here for potential application in gandolinium recovery from medical waste or wastewater.

## Lanthanide drives microbial populations

### https://agu.confex.com/agu/osm20/meetingapp.cgi/Paper/646638

This abstract indicates lanthanide abundance to be an important determinant of methylotroph communities in the Sargasso Sea.

## Microbial protein for recovery of rare earth elements

### https://pubs.acs.org/doi/10.1021/acscentsci.1c01247

This commentary discusses the use of the bacterial protein lanmoduilin (LanM) immobilized on a solid support to bind and recover rare earth metals. The protein has a 100‐million‐fold higher affinity for La^3+^ than Ca^2+^.

## Bicohemistry of lanthanide acquisition

### https://par.nsf.gov/servlets/purl/10200150

This article presents a good overview of lanthanide acquisition, trafficking and utilization by methylotrophic bacteria and their proteins.

## X‐ray structure of rare earth‐containing enzyme

### https://www.rcsb.org/structure/6FKW

This page links to X‐ray crystallographic data and other information on a europium‐containing methanol dehydrogenase.

## Lanthanides and microbes

### https://www.exploringmicrobes.science/pages/research.html

This website provides a good review of the role of lanthanides in methylotrophy.

## Lanthanide homeostasis in a methylotroph

### https://www.nature.com/articles/s41598‐020‐69401‐4

This article discusses lanthanide transport and trafficking genes.

## The lanthanides

### https://www.thoughtco.com/lanthanides‐properties‐606651

This webpage focuses on the general chemical properties and important commercial applications of lanthanide elements.

## Lanthanide biomining projects

### https://arpa‐e.energy.gov/technologies/exploratory‐topics/biomining

This project site of the ARPA‐E program highlights new projects to maintain a robust supply of critical materials for clean energy, including the acquisition of rare earth elements.

## Response to lanthanum in growth of a pseudomonad

### https://journals.asm.org/doi/10.1128/mBio.00516‐20

This paper details a novel route for glycerol metabolism in *Pseudomonas putida* KT2440 induced by the presence of lanthanum.

